# An approach using ddRADseq and machine learning for understanding speciation in Antarctic Antarctophilinidae gastropods

**DOI:** 10.1038/s41598-021-87244-5

**Published:** 2021-04-19

**Authors:** Juan Moles, Shahan Derkarabetian, Stefano Schiaparelli, Michael Schrödl, Jesús S. Troncoso, Nerida G. Wilson, Gonzalo Giribet

**Affiliations:** 1 Museum of Comparative Zoology, Department of Organismic and Evolutionary Biology, Harvard University, 26 Oxford Street, Cambridge, MA 02138 USA; 2 DiSTAV, University of Genoa, C.so Europa 26, 16132 Genoa, Italy; 3 Italian National Antarctic Museum (MNA, Section of Genoa), Viale Benedetto XV n. 5, 16132 Genoa, Italy; 4 Departamento de Ecoloxía e Bioloxía Animal, Universidade de Vigo, Campus Lagoas-Marcosende s/n, 36200 Vigo, Spain; 5 Collections and Research, Western Australian Museum, Welshpool DC, Locked Bag 49, Perth, WA 6986 Australia; 6 School of Biological Sciences, University of Western Australia, 35 Stirling Hwy, Crawley, WA 6009 Australia; 7 Present Address: SNSB-Bavarian State Collection of Zoology, Münchhausenstrasse 21, 81247 Munich, Germany; 8 Present Address: Biozentrum Ludwig Maximilians University and GeoBio-Center LMU Munich, Munich, Germany

**Keywords:** Genomics, Zoology, Phylogenetics, Taxonomy

## Abstract

Sampling impediments and paucity of suitable material for molecular analyses have precluded the study of speciation and radiation of deep-sea species in Antarctica. We analyzed barcodes together with genome-wide single nucleotide polymorphisms obtained from double digestion restriction site-associated DNA sequencing (ddRADseq) for species in the family Antarctophilinidae. We also reevaluated the fossil record associated with this taxon to provide further insights into the origin of the group. Novel approaches to identify distinctive genetic lineages, including unsupervised machine learning variational autoencoder plots, were used to establish species hypothesis frameworks. In this sense, three undescribed species and a complex of cryptic species were identified, suggesting allopatric speciation connected to geographic or bathymetric isolation. We further observed that the shallow waters around the Scotia Arc and on the continental shelf in the Weddell Sea present high endemism and diversity. In contrast, likely due to the glacial pressure during the Cenozoic, a deep-sea group with fewer species emerged expanding over great areas in the South-Atlantic Antarctic Ridge. Our study agrees on how diachronic paleoclimatic and current environmental factors shaped Antarctic communities both at the shallow and deep-sea levels, promoting Antarctica as the center of origin for numerous taxa such as gastropod mollusks.

## Introduction

Traditionally, deep-sea species are regarded as occupying a wide depth range (i.e. eurybathy) and as distributed across large biogeographical areas due to the supposed homogeneity of the deep-sea habitat^1^. However, unlike their shallow-water counterparts, little is known about the evolution and radiation of deep-sea species at a global scale^2–4^. Elucidating the factors that drive diversification in the deep is of profound importance for understanding how deep-sea taxa originated and diversified. Nonetheless, the paucity of taxonomic surveys of deep-sea invertebrates precludes us from having a sound assessment of the diversity and distributional patterns of deep-sea organisms^5,6^. Fossil evidence suggests that many post-Paleozoic taxa first appeared onshore even if they are now exclusive in the deep-sea^7^, and might have been displaced into deeper waters as a result of pressure from predation and/or competition^5^ or due to physical disturbances^8^. There is evidence suggesting that both shallow and deep-sea organisms share a common period of diversification around the Oligocene and Miocene, undoubtedly, due to major tectonic events during these epochs^9–11^.

Antarctica represents an interesting system for comparatively studying shallow *versus* deep water speciation processes. Firstly, low physical disturbance (below the influence of iceberg scour), cold temperatures, and intermittent food availability are shared characteristics for both the Southern Ocean (SO) shelf and deep-sea ecosystems as a whole^12^. Secondly, during the Eocene–Oligocene transition (~ 34 Mya^13^) and due to the effect of the glacial cycles, Antarctica acquired a considerably deeper shelf and slope than other ocean basins^14^. This fact led the Antarctic fauna into the tendency to eurybathy and widespread—often around the continent, i.e. circumpolar—distributions, but reaching the upper and more accessible continental shelf, compared to more typical deep-sea species^15,16^. Concurrently, the onset of the Antarctic Circumpolar Current (ACC) also occurred, and tectonic events leading to the opening of both the Drake and Tasmanian Passages^17^. The ACC connected shallow-water Antarctic fauna with deep water in the Atlantic, Indian, and Pacific Oceans contributing to the Cenozoic diversification in the SO^18,19^. This provides evidence that Antarctica may have acted as a center of origin for deep-sea taxa^20–22^, with its shelf taxa dispersing into deep water using the northward movement of the Antarctic Bottom Water (ABW, 20–5 Mya^18^), as a result of most of the Antarctic continental shelf being covered by grounded ice sheets during glacial periods^17–19^. Nonetheless, the potential ecological or historical mechanisms affecting patterns of spatial and temporal differentiation in Antarctic deep-sea fauna remains largely unexplored.

Notably, the Antarctic deep-sea floor appears to be rich in mollusk gastropod species compared to other ocean plains^23^. Ample evidence has been proposed for the Antarctic origin of two lineages of mollusks, Cephalaspidea and Nudipleura (Gastropoda: Heterobranchia)^24–27^, and dispersal through the deep sea by the ABW^19,22,28^. Thus, a suitable model for studying the origin, diversification, and biogeography of deep-sea organisms and their evolutionary links with shallow water faunas at the SO are cephalaspidean gastropod mollusks, particularly Philinoidea. Recent work on the systematics of philinoid snails from around the world resulted in the division of Philinidae sensu lato into five families^29,30^, including the SO endemic Antarctophilinidae. This family is composed of two genera, the monospecific *Waegelea* with the species *W. antarctica* and *Antarctophiline* with six known species, namely *A. alata*, *A. apertissima*, *A. easmithi*, *A. gibba*, and *A. falklandica* from shelf waters and *A. amundseni* from deeper plains^29^. Although some Antarctophilinidae species are supposed to be circumpolar, encompassing depth ranges from shallow down to 500 m, others appear to have more restricted distributions^29,31^. Antarctophilinid species are endemic to the SO with only rare or dubious records of some species from the adjacent Polar Front boundaries. Both sympatric and allopatric speciation events were described as shaping the family’s diversity with evidence for cryptic or hidden speciation also potentially occurring in this system^29^. Moreover, species records encompass all the Antarctic plateau from shallow to abyssal waters. Thereby, this paper aims to shed light on the origin and diversification in Antarctica by using the radiation of Antarctophilinidae as a case study to ascertain the patterns of diversification across shallow- and deep-water species from the SO and adjacent areas. For that purpose, we used high-throughput genomic data and novel machine learning approaches for unraveling genetic speciation processes in extant species. Moreover, we reevaluate the only known related fossil to further dig into the origin of the family.

## Results

### Phylogenetic reconstruction

**Figure 1:**
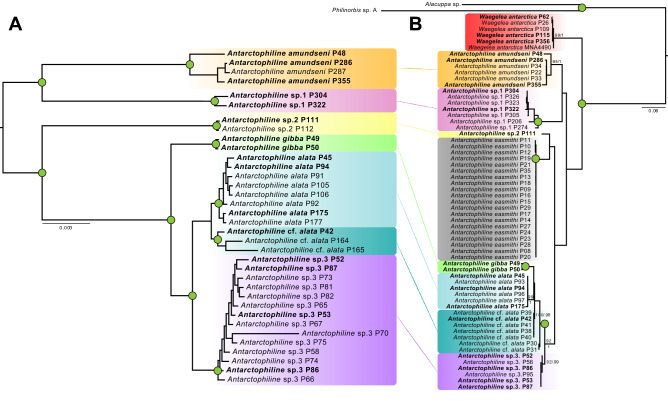
Phylogenetic relationships of antarctophilinids based on maximum likelihood (ML), identical topology was recovered through Bayesian inference (BI), colored boxes illustrating species hypotheses. (**A**) Tree based on ddRADseq data of Matrix 2 (depicted in Figure S1). (**B**) Tree based on COI sequences showing similar clades, but also including the sister group *Waegelea antarctica* in red and *Antarctophiline easmithi* in grey. Green dots denote full support for both bootstrap (ML) and posterior probability values (BI). Samples in bold are present in both trees.

From a total of 142 extracted samples for molecular analyses, only 61 were successfully COI-barcoded and 40 were used in ddRADseq experiments (Table 1, Fig. 1A,B). The sequenced fragment of the COI gene included ca. 658 bp. All newly sequenced samples belong to Antarctophilinidae; the closely related cephalaspideans *Alacuppa* sp. and *Philinorbis* sp. were obtained from GenBank and used as outgroups. The maximum likelihood topology (Fig. 1B) showed the sister group to all *Antarctophiline* species was *Waegelea antarctica* (E. A. Smith, 1902), with samples ranging across the Drake Passage, Ross Sea, Scotia Arc, and Weddell Sea (65–500 m depth). Also, six clades corresponding to *Antarctophiline* species were identified, including *A. easmithi* (E Weddell Sea, 170–460 m depth) not present in the ddRADseq datasets, and depicted in grey in Fig. 1B, and a complex of species with affinity to *A. alata*.

**Table 1:** Samples obtained from the SIO-BIC, MCZ, MNA, WAM, ZMBN (University Museum of Bergen), and ZSM, including voucher numbers, collecting site and date, geographical and bathymetric distribution, and COI barcode, when present.

Species	Code	Voucher number	Barcode	Latitude	Longitude	Gear	Depth (m)	Location	Date	Cruise number	Station number
*W. antarctica* (E. A. Smith, 1902)	P26	ZMBN 121313	MK015702	71° 7.3′ S	11° 28.4′ W	TVG	65	E Weddell Sea	18-Feb-98	ANT XV/3	48/209
***W. antarctica***	**P62**	**SIO-BIC M12655**	**MN486297**	**58° 22.71′ S**	**26° 17′ W**	**BLT**	**134–260**	**N South Sandwich Islands**	**5-Oct-11**	**NBP11-05**	**SS2a/36**
*W. antarctica*	P109	SIO-BIC M13658	MN486298	61° 13′ 3.7″ S	54° 15′ 17.1″ W	BLT	202–223	Elephant Island	22-Oct-11	NBP11-05	EI1/81
***W. antarctica***	**P115**	**SIO-BIC M17788**	**MN486299**	**62° 52**′ **20.7**″ **S**	**57° 11**′ **32.5**″ **W**	**BLT**	**150–247**	**Bransfield Strait**	**24-Oct-11**	**NBP11-05**	**BS1/86**
*W. antarctica*	P117	SIO-BIC M17789	–	62° 52′ 20.7″ S	57° 11′ 32.5″ W	BLT	150–247	Bransfield Strait	24-Oct-11	NBP11-05	BS1/86
*W. antarctica*	P118	SIO-BIC M17790	–	62° 52′ 20.7″ S	57° 11′ 32.5″ W	BLT	150–247	Bransfield Strait	24-Oct-11	NBP11-05	BS1/86
***W. antarctica***	**P356**	**MNA11027**	**MN486300**	**62° 55.99**′ **S**	**58° 40.67**′ **W**	**AGT**	**547**	**Bransfield Strait**	**3-May-13**	**ANT XXIX/3**	**227-2**
*W. antarctica*		MNA 04490	MN486301	74° 45′ 52.2″ S	164° 4′ 55.3″ E	D	100	Adélie Cove, Ross Sea	8-Jan-10	PNRA XXV Exp 09/10	DR4
*A. amundseni* Moles, Avila & Malaquias, 2019	P22	ZMBN 121347	MK015698	73° 36.6′ S	22° 24.7′ W	BT	736	E Weddell Sea	5-Feb-98	ANT XV/3	48/097
*A. amundseni*	P33	ZMBN 121314	MK015708	71° 18.61′ S	13° 56.12′ W	EBS	910	E Weddell Sea	21-Dec-03	ANT XXI/2	PS65/232-1
*A. amundseni*	P34	ZMBN 121348	MK015709	71° 18.61′ S	13° 56.12′ W	EBS	910	E Weddell Sea	21-Dec-03	ANT XXI/2	PS65/232-1
***A. amundseni***	**P48**	**SIO-BIC M13655**	**MN486278**	**55° 4**′ **51.8**″ **S**	**35° 10**′ **21.4**″ **W**	**BLT**	**196–253**	**South Georgia**	**29-Sep-11**	**NBP11-05**	**SG3a/23**
***A. amundseni***	**P286**	**ZSM 27239**	**MN486279**	**71° 18**′ **25.2**″ **S**	**13° 58**′ **13.2**″ **W**	**EBS**	**1048**	**E Weddell Sea**	**20-Feb-05**	**ANT XXII/3**	**PS67/074-6-E**
***A. amundseni***	**P287**	**ZSM 27239**	**–**	**71° 18**′ **25.2**″ **S**	**13° 58**′ **13.2**″ **W**	**EBS**	**1048**	**E Weddell Sea**	**20-Feb-05**	**ANT XXII/3**	**PS67/074-6-E**
***A. amundseni***	**P355**	**MNA 11026**	**MN486280**	**62° 55.99**′ **S**	**58° 40.67**′ **W**	**AGT**	**547**	**Bransfield Strait**	**3-May-13**	**ANT XXIX/3**	**227-2**
*Antarctophiline* sp. 1	P206	ZSM 20854	MN486283	62° 57′ 48″ S	27° 52′ 8.4″ W	AGT	4548	Weddell Sea—S South Sandwich Islands	16-Mar-02	ANT XIX/4	PS61/138-4
*Antarctophiline* sp. 1	P274	ZSM 21093	MN486284	60° 39′ 11.4″ S	53° 56′ 51″ W	EBS	2893	N Elephant Island	30-Jan-02	ANT XIX	PS61/046-7
***Antarctophiline*** **sp.** **1**	**P304**	**ZSM 34346**	**MN486285**	**52° 2**′ **31.8**″ **S**	**0° 0**′ **36**″ **E**	**AGT**	**2996**	**NW Bouvet Island, S Atlantic Ocean**	**6-Dec-07**	**ANT XXIV/2**	**PS71/013-15**
*Antarctophiline* sp. 1	P305	ZSM 34346	MN486286	52° 2′ 31.8″ S	0° 0′ 36″ E	AGT	2996	NW Bouvet Island, S Atlantic Ocean	6-Dec-07	ANT XXIV/2	PS71/013-15
***Antarctophiline*** **sp.** **1**	**P322**		**MN486287**	**52° 0.36**′ **S**	**10° 1.47**′ **E**	**EBS**	**3705–3757**	**NW Bouvet Island, S Atlantic Ocean**	**20-Jan-12**	**ANT XXVIII/3**	**PS79/081-18**
*Antarctophiline* sp. 1	P323		MN486288	52° 0.18′ S	10° 0.72′ E	EBS	3743–3763	NE Bouvet Island, S Atlantic Ocean	20-Jan-12	ANT XXVIII/3	PS79/081-17
*Antarctophiline* sp. 1	P326		MN486289	52° 0.18′ S	10° 0.72′ E	EBS	3743–3763	NE Bouvet Island, S Atlantic Ocean	20-Jan-12	ANT XXVIII/3	PS79/081-17
***Antarctophiline*** **sp.** **2**	**P111**	**SIO-BIC M17786**	**MN486290**	**62° 52**′ **20.7**″ **S**	**57° 11**′ **32.5**″ **W**	**BLT**	**150–247**	**Bransfield Strait**	**24-Oct-11**	**NBP11-05**	**BS1/86**
***Antarctophiline*** **sp.** **2**	**P112**	**SIO-BIC M17787**	**–**	**62° 52**′ **20.7**″ **S**	**57° 11**′ **32.5**″ **W**	**BLT**	**150–247**	**Bransfield Strait**	**24-Oct-11**	**NBP11-05**	**BS1/86**
*A. easmithi* Moles, Avila & Malaquias, 2019	P08	ZMBN 121327	MK015684	71° 18.6′ S	12° 18.1′ W	AGT	173	E Weddell Sea	25-Jan-98	ANT XV/3	PS48/006
*A. easmithi*	P09	ZMBN 121328	MK015685	71° 18.6′ S	12° 18.1′ W	AGT	173	E Weddell Sea	25-Jan-98	ANT XV/3	PS48/006
*A. easmithi*	P10	ZMBN 121329	MK015686	71° 19.3′ S	12° 24.7′ W	TVG	182	E Weddell Sea	28-Jan-98	ANT XV/3	PS48/027
*A. easmithi*	P11	ZMBN 121330	MK015687	70° 52.7′ S	10° 34.8′ W	AGT	230	E Weddell Sea	30-Jan-98	ANT XV/3	PS48/044
*A. easmithi*	P12	ZMBN 121331	MK015688	70° 52.7′ S	10° 34.8′ W	AGT	230	E Weddell Sea	30-Jan-98	ANT XV/3	PS48/044
*A. easmithi*	P13	ZMBN 121332	MK015689	70° 52.7′ S	10° 34.8′ W	AGT	230	E Weddell Sea	30-Jan-98	ANT XV/3	PS48/044
*A. easmithi*	P14	ZMBN 121333	MK015690	70° 52.7′ S	10° 34.8′ W	AGT	230	E Weddell Sea	30-Jan-98	ANT XV/3	PS48/044
*A. easmithi*	P15	ZMBN 121334	MK015691	70° 54′ S	10° 28.2′ W	AGT	232	E Weddell Sea	31-Jan-98	ANT XV/3	PS48/062
*A. easmithi*	P16	ZMBN 121335	MK015692	70° 54′ S	10° 28.2′ W	AGT	232	E Weddell Sea	31-Jan-98	ANT XV/3	PS48/062
*A. easmithi*	P17	ZMBN 121336	MK015693	70° 54′ S	10° 28.2′ W	AGT	232	E Weddell Sea	31-Jan-98	ANT XV/3	PS48/062
*A. easmithi*	P18	ZMBN 121337	MK015694	72° 51.7′ S	19° 7.9′ W	BT	439	E Weddell Sea	3-Feb-98	ANT XV/3	PS48/078
*A. easmithi*	P19	ZMBN 121338	MK015695	72° 50.5′ S	19° 28′ W	BT	463	E Weddell Sea	3-Feb-98	ANT XV/3	PS48/082
*A. easmithi*	P20	ZMBN 121339	MK015696	72° 50.5′ S	19° 28′ W	BT	463	E Weddell Sea	3-Feb-98	ANT XV/3	PS48/082
*A. easmithi*	P21	ZMBN 121340	MK015697	72° 50.5′ S	19° 28′ W	BT	463	E Weddell Sea	3-Feb-98	ANT XV/3	PS48/082
*A. easmithi*	P23	ZMBN 121341	MK015699	73° 39.1′ S	20° 59.6′ W	D	211	E Weddell Sea	8-Feb-98	ANT XV/3	PS48/128
*A. easmithi*	P24	ZMBN 121342	MK015700	73° 39.1′ S	20° 59.6′ W	D	211	E Weddell Sea	8-Feb-98	ANT XV/3	PS48/128
*A. easmithi*	P27	ZMBN 121343	MK015703	70° 50.5′ S	10° 41.8′ W	BT	307	E Weddell Sea	19-Feb-98	ANT XV/3	PS48/222
*A. easmithi*	P28	ZMBN 121344	MK015704	71° 18′ S	12° 15′ W	AGT	184	E Weddell Sea	27-Feb-98	ANT XV/3	PS48/277
*A. easmithi*	P29	ZMBN 121345	MK015705	71° 18′ S	12° 15′ W	AGT	184	E Weddell Sea	27-Feb-98	ANT XV/3	PS48/277
*A. easmithi*	P35	ZMBN 121346	MW509525	71° 04.30′ S	11° 33.92′ W	BT	309	E Weddell Sea	23-Dec-03	ANT XXI/2	PS65/253-1
***A. gibba*** **(Strebel,** **1908)**	**P49**	**SIO-BIC M12896**	**MN486281**	**55° 2**′ **S**	**35° 26**′ **W**	**BLT**	**125**	**South Georgia**	**29-Sep-11**	**NBP11-05**	**SG3/22**
***A. gibba***	**P50**	**SIO-BIC M12896**	**MN486282**	**55° 2**′ **S**	**35° 26**′ **W**	**BLT**	**125**	**South Georgia**	**29-Sep-11**	**NBP11-05**	**SG3/22**
***A. alata*** **(Thiele,** **1912)**	**P45**	**WAMS101214**	**MN486272**	**59° 28**′ **11.3**″ **S**	**27° 16**′ **44.8**″ **W**	**AGT**	**230**	**Southern Thule, South Sandwich Islands**	**8-Mar-17**	**ACE2016-17**	**90/2590**
***A. alata***	**P91**	**SIO-BIC M17793**	**–**	**59° 23**′ **40.8**″ **S**	**27° 18**′ **41.7**″ **W**	**BLT**	**103–221**	**S South Sandwich Islands**	**7-Oct-11**	**NBP11-05**	**SS3/42**
***A. alata***	**P92**	**SIO-BIC M17794**	**–**	**59° 23**′ **40.8**″ **S**	**27° 18**′ **41.7**″ **W**	**BLT**	**103–221**	**S South Sandwich Islands**	**7-Oct-11**	**NBP11-05**	**SS3/42**
*A. alata*	P93	SIO-BIC M17795	MN486273	59° 23′ 40.8″ S	27° 18′ 41.7″ W	BLT	103–221	S South Sandwich Islands	7-Oct-11	NBP11-05	SS3/42
***A. alata***	**P94**	**SIO-BIC M13654**	**MN486274**	**59° 23**′ **40.8**″ **S**	**27° 18**′ **41.7**″ **W**	**BLT**	**103–221**	**S South Sandwich Islands**	**7-Oct-11**	**NBP11-05**	**SS3/42**
*A. alata*	P96	SIO-BIC M17796	MN486275	59° 23′ 11.4″ S	27° 18′ 49.2″ W	BLT	403–501	S South Sandwich Islands	8-Oct-11	NBP11-05	SS3/44
*A. alata*	P97	SIO-BIC M17797	MN486276	59° 23′ 11.4″ S	27° 18′ 49.2″ W	BLT	403–501	S South Sandwich Islands	8-Oct-11	NBP11-05	SS3/44
***A. alata***	**P105**	**SIO-BIC M17798**	**–**	**59° 23**′ **11.4**″ **S**	**27° 18**′ **49.2**″ **W**	**BLT**	**403–501**	**S South Sandwich Islands**	**8-Oct-11**	**NBP11-05**	**SS3/44**
***A. alata***	**P106**	**SIO-BIC M17799**	**–**	**59° 23**′ **11.4**″ **S**	**27° 18**′ **49.2**″ **W**	**BLT**	**403–501**	**S South Sandwich Islands**	**8-Oct-11**	**NBP11-05**	**SS3/44**
***A. alata***	**P175**	**ZSM 15955**	**MN486277**	**70° 50**′ **12**″ **S**	**10° 35**′ **24**″ **W**	**BT**	**271**	**E Weddell Sea**	**4-Oct-00**	**ANT XVII/3**	**136-1**
***A. alata***	**P177**	**ZSM 15955**	**–**	**70° 50**′ **12**″ **S**	**10° 35**′ **24**″ **W**	**BT**	**271**	**E Weddell Sea**	**4-Oct-00**	**ANT XVII/3**	**136-1**
*A.* cf*. alata*	P30	ZMBN 121350	MK015706	54° 30.01′ S	3° 13.97′ E	AGT	260	Bouvet Island	24-Nov-03	ANT XXI/2	PS65/019-1
*A*. cf. *alata*	P31	ZMBN 121351	MK015707	54° 22.49′ S	3° 17.58′ E	AGT	134	Bouvet Island	24-Nov-03	ANT XXI/2	PS65/028-1
*A.* cf*. alata*	P38	ZMBN 121323	MK015710	62° 58.18S	60° 42.23 W	SD	10	Fumarole Bay, Deception Island	25-Jan-13	ACTIQUIM-4	A4-453
*A.* cf*. alata*	P39	ZMBN 121324	MK015711	62° 58.18S	60° 42.23 W	SD	10	Fumarole Bay, Deception Island	25-Jan-13	ACTIQUIM-4	A4-453
*A.* cf*. alata*	P40	ZMBN 121315	MK015712	62° 58.18S	60° 42.23 W	SD	10	Fumarole Bay, Deception Island	25-Jan-13	ACTIQUIM-4	A4-453
*A.* cf*. alata*	P41	ZMBN 121325	MK015713	62° 58.18S	60° 42.23 W	SD	10	Fumarole Bay, Deception Island	25-Jan-13	ACTIQUIM-4	A4-453
***A.*** **cf.** ***alata***	**P42**	**ZMBN 121326**	**MK015714**	**62° 58.18S**	**60° 42.23 W**	**SD**	**10**	**Fumarole Bay, Deception Island**	**25-Jan-13**	**ACTIQUIM-4**	**A4-453**
***A.*** **cf.** ***alata***	**P164**	**MCZ 393955**	**–**	**62° 40**′ **S**	**60° 38**′ **W**	**RD**	**216**	**S Livingston Island, South Shetlands Islands**	**19-Feb-94**	**BENTART-95**	**100R**
***A.*** **cf.** ***alata***	**P165**	**MCZ 393955**	**–**	**62° 40**′ **S**	**60° 38**′ **W**	**RD**	**216**	**S Livingston Island, South Shetlands Islands**	**19-Feb-94**	**BENTART-95**	**100R**
***Antarctophiline*** **sp.** **3**	**P52**	**SIO-BIC M13656**	**MN486291**	**56° 42**′ **50.6**″ **S**	**27° 1**′ **35.8**″ **W**	**BLT**	**134–142**	**N South Sandwich Islands**	**3-Sep-11**	**NBP11-05**	**SS1A/30**
***Antarctophiline*** **sp.** **3**	**P53**	**SIO-BIC M12975**	**MN486292**	**58° 28.1**′ **S**	**26° 13.1**′ **W**	**BLT**	**164–172**	**N South Sandwich Islands**	**5-Oct-11**	**NBP11-05**	**SS2/34**
*Antarctophiline* sp. 3	P56	SIO-BIC M17800	MN486293	58° 22′ S	26° 16′ W	BLT	153–420	N South Sandwich Islands	5-Oct-11	NBP11-05	SS2a/36
***Antarctophiline*** **sp.** **3**	**P58**	**SIO-BIC M17801**	**–**	**58° 22**′ **S**	**26° 16**′ **W**	**BLT**	**153–420**	**N South Sandwich Islands**	**5-Oct-11**	**NBP11-05**	**SS2a/36**
***Antarctophiline*** **sp.** **3**	**P65**	**SIO-BIC M17802**	**–**	**58° 22.71**′ **S**	**26° 17**′ **W**	**BLT**	**134–260**	**N South Sandwich Islands**	**6-Oct-11**	**NBP11-05**	**SS2a/38**
***Antarctophiline*** **sp.** **3**	**P66**	**SIO-BIC M17803**	**–**	**58° 22.71**′ **S**	**26° 17**′ **W**	**BLT**	**134–260**	**N South Sandwich Islands**	**6-Oct-11**	**NBP11-05**	**SS2a/38**
***Antarctophiline*** **sp.** **3**	**P67**	**SIO-BIC M17804**	**–**	**58° 22.71**′ **S**	**26° 17**′ **W**	**BLT**	**134–260**	**N South Sandwich Islands**	**6-Oct-11**	**NBP11-05**	**SS2a/38**
***Antarctophiline*** **sp.** **3**	**P70**	**SIO-BIC M17805**	**–**	**58° 22.71**′ **S**	**26° 17**′ **W**	**BLT**	**134–260**	**N South Sandwich Islands**	**6-Oct-11**	**NBP11-05**	**SS2a/38**
***Antarctophiline*** **sp.** **3**	**P73**	**SIO-BIC M17806**	**–**	**58° 22.71**′ **S**	**26° 17**′ **W**	**BLT**	**134–260**	**N South Sandwich Islands**	**6-Oct-11**	**NBP11-05**	**SS2a/38**
***Antarctophiline*** **sp.** **3**	**P74**	**SIO-BIC M17807**	**–**	**58° 22.71**′ **S**	**26° 17**′ **W**	**BLT**	**134–260**	**N South Sandwich Islands**	**6-Oct-11**	**NBP11-05**	**SS2a/38**
***Antarctophiline*** **sp.** **3**	**P75**	**SIO-BIC M17808**	**–**	**58° 22.71**′ **S**	**26° 17**′ **W**	**BLT**	**134–260**	**N South Sandwich Islands**	**6-Oct-11**	**NBP11-05**	**SS2a/38**
***Antarctophiline*** **sp.** **3**	**P81**	**SIO-BIC M17809**	**–**	**58° 22.71**′ **S**	**26° 17**′ **W**	**BLT**	**134–260**	**N South Sandwich Islands**	**6-Oct-11**	**NBP11-05**	**SS2a/38**
***Antarctophiline*** **sp.** **3**	**P82**	**SIO-BIC M17810**	**–**	**58° 22.71**′ **S**	**26° 17**′ **W**	**BLT**	**134–260**	**N South Sandwich Islands**	**6-Oct-11**	**NBP11-05**	**SS2a/38**
***Antarctophiline*** **sp.** **3**	**P86**	**SIO-BIC M17811**	**MN486294**	**58° 22.71**′ **S**	**26° 17**′ **W**	**BLT**	**134–260**	**N South Sandwich Islands**	**6-Oct-11**	**NBP11-05**	**SS2a/38**
***Antarctophiline*** **sp.** **3**	**P87**	**SIO-BIC M17812**	**MN486295**	**58° 22.71**′ **S**	**26° 17**′ **W**	**BLT**	**134–260**	**N South Sandwich Islands**	**6-Oct-11**	**NBP11-05**	**SS2a/38**
*Antarctophiline* sp. 3	P95	SIO-BIC M13093	MN486296	59° 23.19′ S	27° 18.82′ W	BLT	403–501	N South Sandwich Islands	8-Oct-11	NBP11-05	SS3/44

Specimens in bold were included in the ddRADseq phylogenetic analysis. Gear types: *AGT* Agassiz trawl, *BLT* Blake trawl, *BT* bottom trawl, *D* rock dredge, *ES* epibenthic sled, *RD* Rauschert dredge, *SD* SCUBA diving, *TVG* TV grab.

The initial ddRADseq Matrix 1 including both antarctophilinid genera (i.e. *Antarctophiline* and *Waegelea*) included 40 individuals, 3893 loci, and 38.5% missing data (not depicted; see “Methods” section). The outgroup included five individuals of *W. antarctica*, a species with a circumpolar distribution. Matrix 2 was built to increase the number of loci and resolution of the tree, including only the 35 individuals in the genus *Antarctophiline*. The final Matrix 2 dataset contained 5411 loci and 41.6% missing data (see summary statistics in Supplementary Table S1 and plotted matrix visualization in Fig. S1). A particularly long branch was found in sample P70 (Fig. 1A), most likely due to cross-contamination and/or high levels of missing data (see the section below). The subset to specifically address the *A. gibba*/*A. alata* species complex includes 27 individuals (Matrix 3), 5411 loci, and 41.6% missing data. Although several clustering thresholds were tested, only a 75% threshold of similarity produced an output to construct a matrix. This is due to the high genetic divergences among species, and thus, the high amount of singleton reads that makes the clustering within and across samples computationally too demanding. The ML tree of Matrix 2 was rooted with *A. amundseni* Moles, Avila & Malaquias, 2019 plus the abyssal *Antarctophiline* sp. 1 (Fig. 1A) as the sister group to the rest of *Antarctophiline* species. This was also found in the tree of Matrix 1 (Fig. S2), for which an identical topology and maximum bootstrap support (BS = 100) and posterior probability (PP = 1) values were recovered for most nodes, but sample P70 due to potential contamination.

### Distinctiveness in genetic lineages

**Figure 2:**
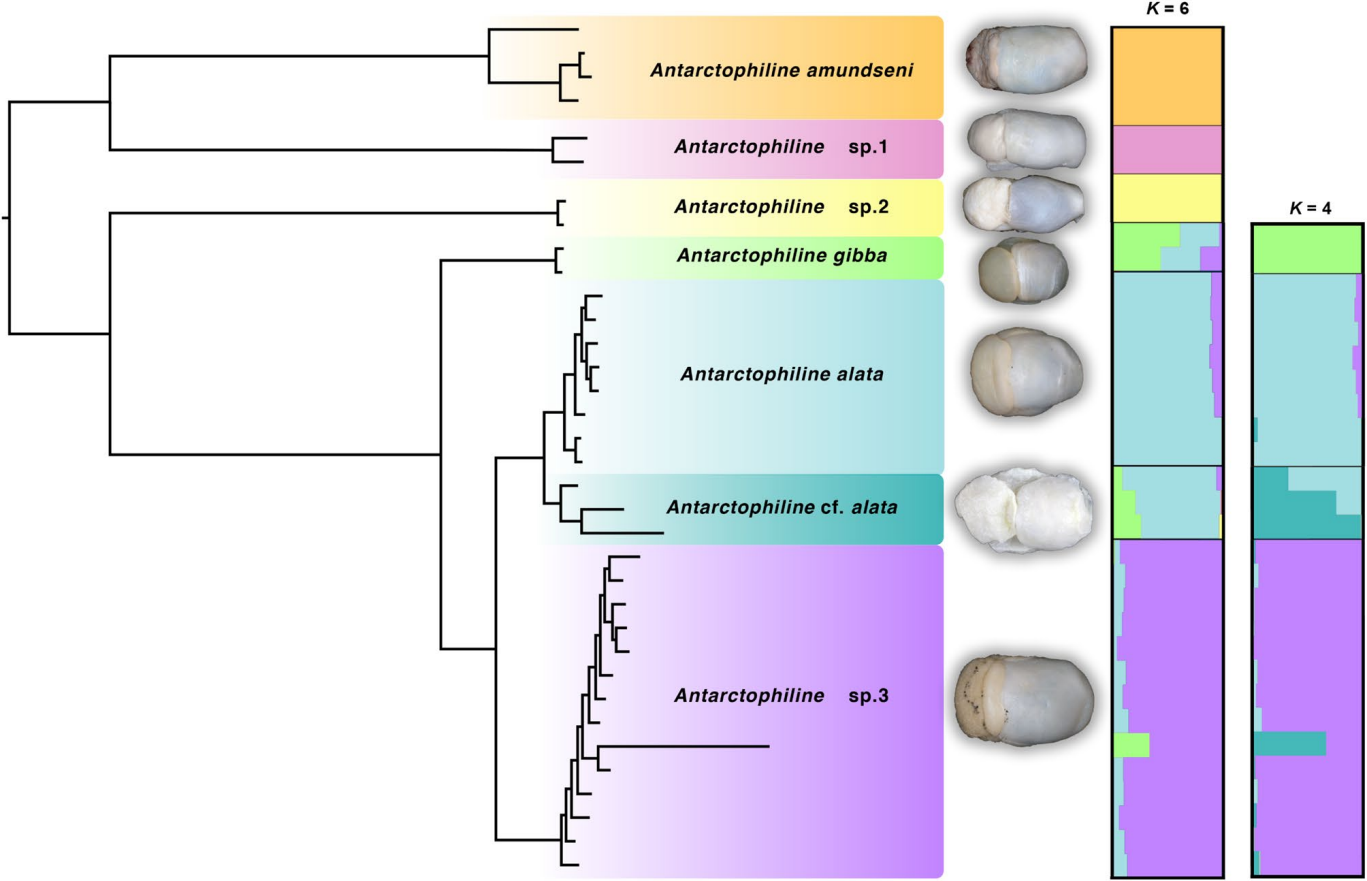
Phylogram of antarctophilinids based on Matrix 2 (left) and STRUCTURE plots (right), showing the posterior probability for individual assignments of samples to different genetic clusters. Both plots show the result for the most likely number of genetic clusters for Matrix 2 (*K* = 6) and Matrix 3 (*K* = 4). Dorsal pics of preserved type specimens for each cluster are also depicted.

For Matrix 2, STRUCTURE (Evanno method^32^) favored an optimal *K* = 6 (Fig. 2), recovering all six a priori barcoded *Antarctophiline* species as distinct clusters, including the eurybathic *A. amundseni* (South Georgia, 200 m depth; Bransfield Strait, 550 m depth; E Weddell Sea, 740–1050 m depth), the abyssal *Antarctophiline* sp. 1 (Scotia Sea, Weddell Sea, NW Bouvet Island, 2900–4500 m depth), a shallow-water *Antarctophiline* sp. 2 (Bransfield Strait, 200 m depth), and distinct clades of the *A. gibba*/*A. alata* species complex not recovered in the barcode phylogeny (including *Antarctophiline* sp. 3). For Matrix 3, STRUCTURE retrieved an optimal *K* = 4 (Fig. 2), clearly splitting *A. gibba* (South Georgia, 125 m depth) from three potentially cryptic shallow-water species with affinity to *A. alata* tentatively named: *A. alata* (S of the South Sandwich Islands plus E Weddell Sea, 100–500 m depth), *A.* cf. *alata* (South Shetland Islands, 10–200 m depth), and *Antarctophiline* sp. 3 (N of the South Sandwich Islands, 130–500 m depth)*.*

**Figure 3:**
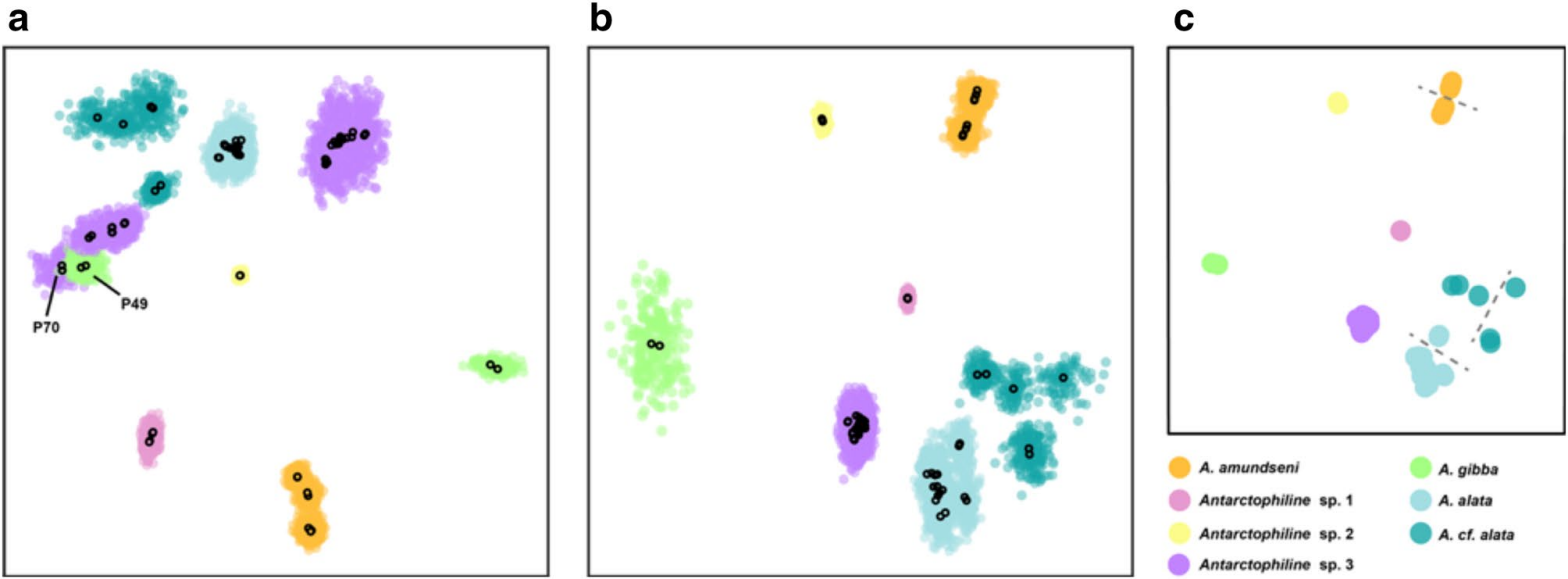
Variational autoencoder (VAE) showing two haplotypes (circles) per sample and clustering results. (**A**) VAE output on the full dataset with a mean (black outlined circle) and standard deviation (colored circles) for each sample. (**B**) VAE output on the dataset with P49 and P70 removed. (**C**) Results of PAM and hierarchical clustering analyses on the VAE output of the dataset with P49 and P70 removed, favoring seven clusters. Dashed lines indicate further split clusters recovered with the gap statistic.

Clustering with the VAE output of the full dataset resulted in 8–10 clusters (Fig. 3A). However, examining the VAE plot, it is apparent that samples P70 and P49 were misplaced and likely confounded an accurate representation of the data, for example, showing two widely divergent *Antarctophiline* sp. 3 clusters. As previously mentioned, P70 likely contains contamination and was removed from subsequent analyses. Additionally, the placement of P49 with *Antarctophiline* sp. 3 (specifically with P70) instead of the other *A. gibba* (P50) is perhaps driven by a combination of admixture (Fig. 1A) and high levels of missing data (up to 80%, see Fig. S1) in this sample, and was also removed. VAE output of the dataset with these two samples removed (Fig. 3B) recovers a single cluster for all *Antarctophiline* sp. 3 samples, more in line with the results from COI-barcoding and ddRADseq analyses. “Partition around medoids” (PAM) and hierarchical clustering on this VAE output recover seven clusters (Fig. 3C), while the gap statistic favors 10 clusters, splitting *A. alata*, *A.* cf. *alata*, and *A. amundseni* into two clusters each. The gap statistic is thus probably over-splitting these taxa as overlapping VAE standard deviations indicate one cluster for *A. amundseni* and at most two clusters for *A.* cf. *alata* (Fig. 3B). Given the concordance between genetic clustering across multiple approaches, we favor seven species, as shown in the VAE clustering (Fig. 3B) results that match those in the phylogeny (Fig. 1) and the sum of the STRUCTURE analyses (Fig. 2).

### Fossil systematic reassessment

- Class Gastropoda Cuvier, 1795
- Order Cephalaspidea Fischer, 1883
- Superfamily Philinoidea Gray, 1850 (1815)
- Family Antarctophilinidae Moles, Avila & Malaquias, 2019

**Figure 4:**
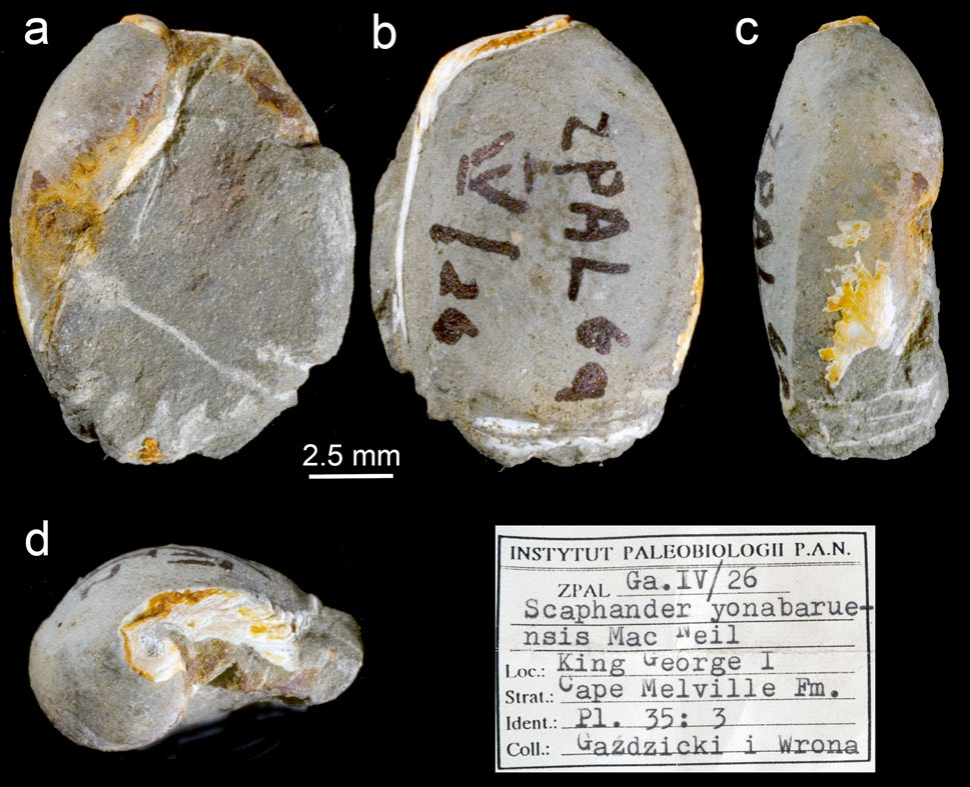
Images of the single fossil of Philinoidea found in Antarctica (King George Island) dated from the Lower Miocene and originally attributed to the Japanese *Scaphander yonabaruensis* Mac Neil, 1960 (Karczewski, 1987), but here redesignated to Antarctophilinidae. (**a**) Ventral view. (**b**) Dorsal view. (**c**) Lateral view. (**d**) Apical view.

*Material examined* (Fig. 4). King George Island, Melville Peninsula (Crab Creek locality, I), Cape Melville Formation, Lower Miocene: 1 specimen, ZPAL Ga. IV/26, length = 11 mm, width = 5 mm.

*Diagnosis* Shell ovate-subquadrate, slightly flattened dorsoventrally; aperture wide; apex obtuse, slightly sunken; outer lip convex; posterior edge of outer lip obtuse, not protruding beyond apex; columellar wall concave; growth lines visible.

*Remarks* Although originally attributed to *Scaphander yonabaruensis* Mac Neil, 1960 known from the Miocene of Japan, in Okinawa^33^, Beu^34^ suggested its similarity to the genus *Philine.* Indeed, we believe the overall shell morphology matches the recently erected family Antarctophilinidae which epitomizes most Philinoidea diversity known from Antarctic waters.

## Discussion

This study, grounded on a large collecting effort in remote and abyssal areas in the SO aided by phylogenetic analyses using ddRADseq-derived SNP data, increases our understanding of Antarctic benthic gastropod species distributions and diversity. Our barcode and STRUCTURE analyses served as a starting point for discerning among genetic lineages^35^. Since incongruences in the number of distinct genetic groups recovered in both Matrix 2 and 3 were found using STRUCTURE, we used novel unsupervised machine learning methods^36^. VAEs proved to be powerful and resolutive when using genomic data for recovering congruent genetic lineages across methods, some of which correspond to species hypothesis. Our results corroborated the latest systematic assessment by Moles et al.^29^ on species diversity and support the likelihood of further cryptic diversity in Antarctophilinidae. Out of the eight delimited species three are considered undescribed, the abyssal *Antarctophiline* sp. 1, the shallow-water species *Antarctophiline* sp. 2 from Bransfield Strait, and *Antarctophiline* sp. 3 from the South Sandwich Islands. Regarding *A*. cf. *alata* and, to a certain extent, *Antarctophiline* sp. 3, these are considered to be at the early stages of speciation (potential cryptic species). Taxonomic descriptions of the undescribed species, as well as the validity of certain synonymized taxa within the *A. alata* species complex (*Philine gouldi* Doello-Jurado, 1918, and *P. amoena* Thiele, 1925), will follow in a separate manuscript. Ecological restrictions to bottom-dwelling habitats may be a driver for the morphological convergence in species of Philinoidea^29,30,37^. Here, genomic data have enhanced the phylogenetic resolution of Antarctophilinidae species obtained through Sanger analysis, particularly for less diverged species, an approach that has been previously applied only to a handful of Antarctic samples (e.g., Ref. ^38^).

A total of seven species of *Antarctophiline* are found in Antarctic shallow waters, the five species analyzed here: *A. alata*, *A. easmithi*, *A. gibba*, *Antarctophiline* sp. 2, and *Antarctophiline* sp. 3 from the vicinities of the Scotia Arc and western Antarctic Peninsula plus the species from the Ross Sea *A. apertissima* (E. A. Smith, 1902) and *A. falklandica* (Powell, 1951) (also from the Falkland Islands^39,40^). Contrary, the deep-sea fauna is less diverse, with only two species known to date. Independent colonization of the continental shelf from the slope during interglacial cycles, < 23 Mya^14,41^, and the presence of habitat refugia in the Antarctic Peninsula tip and adjacent islands^42,43^ may explain the high species endemism and richness found in this study—but this remains to be tested. This phenomenon has been referred to as the Antarctic Biodiversity Pump^21,44^ and sustains habitat fragmentation during glacial maxima as the driving force towards allopatric speciation. Secondly, the present island patchiness across the Scotia Arc (from South Georgia to the South Shetland Islands) may have allowed for rapid radiation and speciation processes due to the availability of different ecological niches^38,45,46^. This could explain the relatively restricted distributions found for *A. gibba*, *Antarctophiline* sp. 2, and *Antarctophiline* sp. 3. Additionally, high species richness is expected due to the elevated productivity of these shallow waters during warm seasons^41^, a pivotal influence controlling Antarctic benthic diversity^47^. In fact, *A. gibba* is endemic to South Georgia, an island considered a hotspot for gastropod diversity, with more than 50 endemic species recorded^48^*.* Nonetheless, the high degree of single species found at each collecting site underlines the difficulty of gathering data on Antarctic ecosystems, and thus, conclusions should be made with caution, especially concerning deep-sea samples^49^. Overall, our study provides evidence for high diversity in a group of species previously considered to be rather low, which may be the result of fluctuating paleoclimatic history and current habitat heterogeneity.

Antarctica has long been considered a center of radiation of marine benthic taxa^19–21^, and heterobranch gastropods in particular^24,25,27^. Here, the single fossil record from the Oligocene–Early Miocene at King George Island is attributed to Antarctophilinidae (Fig. 4), proposing that these snails were present in shallow waters of the South Shetland Islands at least 20 Mya. Although limited water transport is hypothesized during the Eocene 50–34 Mya, and probably until the mid-Miocene 15 Mya^28,50^, Trans-Antarctic migrations through a Ross*-*Weddell seaway through the Amundsen Sea could partially explain the disjunct distributions of several shallow-water gastropods^41,51^. In our study, molecular data for *W. antarctica* supports this disjunct distribution and, the specimens found in Peter I Island—an intermediate locality in the Bellingshausen Sea—further reinforce this hypothesis (reexamined material from Aldea & Troncoso^52^). This has been suggested for other marine benthic taxa^50,53^, for which a circumpolar distribution seems unlikely, but instead, a disjunct distribution has been documented. Our compiling evidence suggests that the Scotia Arc and the Weddell Sea have played a pivotal role in the evolution and radiation of many molluscan species from the Miocene forward. However, until comprehensive sampling has been carried out—something difficult to accomplish in Antarctica—our understanding of species distributions remains somewhat limited.

**Figure 5:**
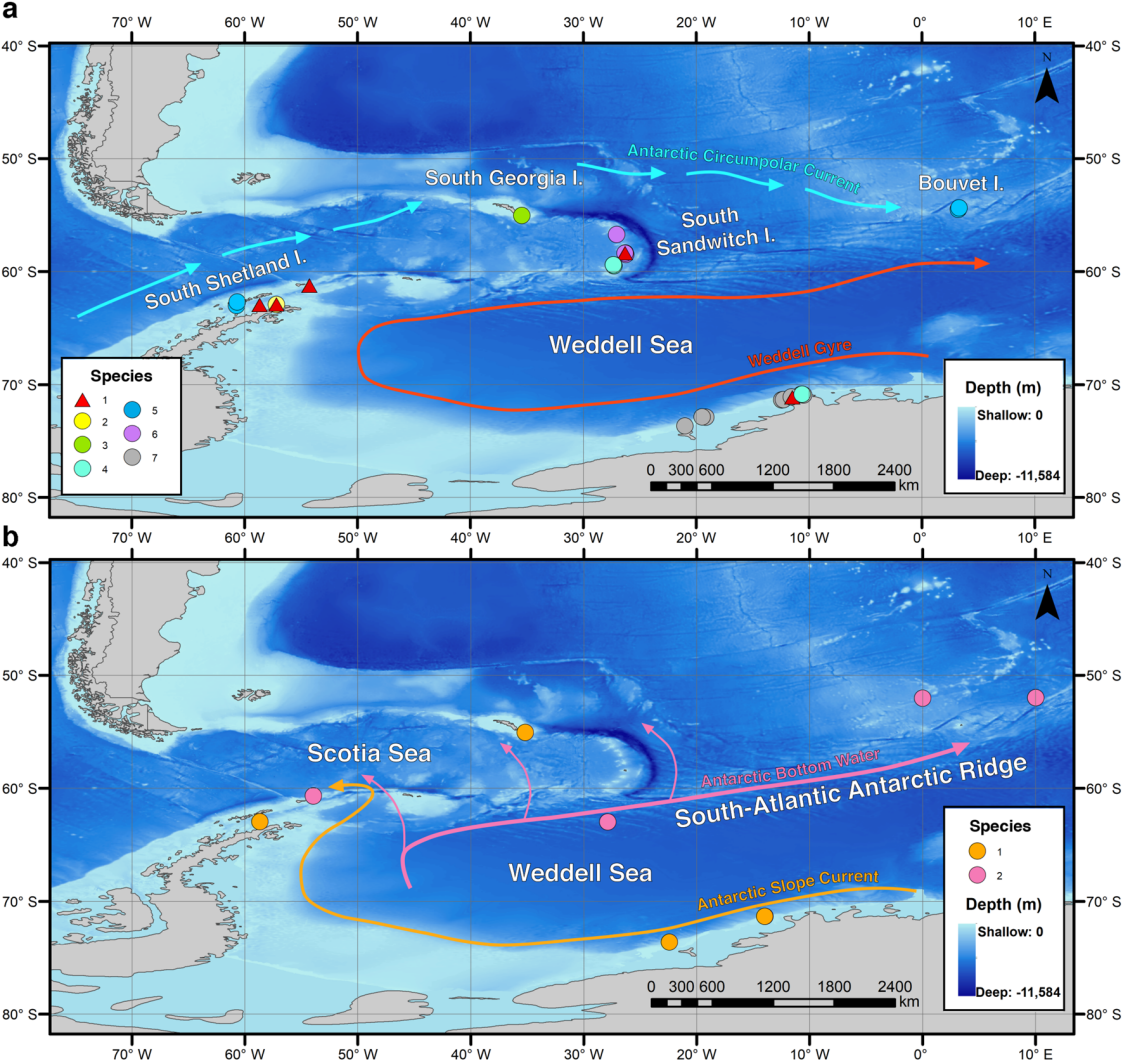
Bathymetric maps showing the distribution of the antarctophilinid specimens from the Atlantic sector of the Southern Ocean including the Scotia Sea, Eastern Weddell Sea, and South Atlantic. (**a**) Shallow water species: (1) *Waegelea antarctica*; (2) *Antarctophiline* sp. 2; (3) *A. gibba*; (4) *A. alata*; (5) *A.* cf. *alata*; (6) *Antarctophiline* sp. 3; (7) *A. easmithi*. (**b**) Deep-sea species: (1) *A. amundseni*; (2) *Antarctophiline* sp. 1.

The onset of the ACC led to the isolation of the Antarctic continent and subsequent cool down with the likely extinction of shallow-water faunas^17^. During interglacial periods of shelf ice retreat, the unpopulated shelf could have been re-colonized by fauna from the slope^54^ or shelters on the continental shelf^14,42,43^. Species dispersal and gene flow at subtidal and shelf depths have been increasingly studied in SO areas with enough evidence of contrasting patterns related to the disparity in species life histories^55,56^, usually challenging the concept of well-connected, circumpolar distributions^57–59^ but see Moore et al.^60^. Extensive geographical distributions were found in species such as *A*. *alata* (including *A*. cf. *alata*) and *W. antarctica,* which occur at 10–500 m depth over the Eastern Weddell Sea, the South Shetland Islands, and the Southern South Sandwich Islands, even extending towards the more remote Bouvet Island. The Weddell Gyre is a clockwise current known for connecting shallow shelf waters along the Weddell Sea coast to the South Shetland Islands (through the tip of the Antarctic Peninsula), and towards the South Sandwich Islands, which might ultimately reach Bouvet Island^61,62^. Dispersal through this hydrographic jet could explain the current distribution among the studied species (see Fig. 5a). The Scotia Arc faunal gateway, helped by oceanic eddies, might ultimately be responsible for the distribution found across the Sub-Antarctic islands of South Sandwich and South Georgia^63,64^. Unfortunately, scarce is information on the life history of Antarctophilinidae. The studied species *A. gibba* lays egg masses at the superficial waters of South Georgia, these containing thousands of large eggs lacking a planktonic phase^65^. Just recently, the larval development of *W. antarctica* was studied in detail showing early juvenile stages hatching with a relict larval velum, being able to drift away in water currents^66^. We hypothesize this is a drifting mode that may explain our current knowledge of distribution in this family (see Ref. ^67^).

Cenozoic glacial-interglacial cycles may have also represented the environmental force that shaped the evolutionary trend toward eurybathy in many Antarctic benthic invertebrates^14,54,68^. During periods of extension of the continental ice sheet, an *Antarctophiline* lineage from the shelf may have been forced to go into deep slope refugia. In this sense, the sister group to the shallow water *Antarctophiline* clade is a deep-sea group composed of the recently described *A. amundseni* and an undescribed abyssal species, both displaying a bathymetrically separated distribution but with widespread geographical distributions. At the upper bathyal zone, which includes the Antarctic slope, we found populations of *A. amundseni* across the Eastern Weddell Sea and the Bransfield Strait. The Antarctic Slope Current circulating in a counterclockwise direction could have been responsible for such distributions^69^. Far below, very distant populations of *Antarctophiline* sp. 1 at 2900–4500 m depth were recorded west of the Antarctic Peninsula, over the Scotia Ridge, and towards the northern and eastern regions of Bouvet Island. The distribution expands through the South-Atlantic Antarctic Ridge (Fig. 5b), strikingly covering a linear distance of more than 3900 km. The eastern jet of the ABW (i.e., Weddell Sea Bottom Water) feeds the Atlantic abyssal waters all over the North Atlantic as part of the Global Thermohaline Circulation^70–72^ and indeed reflects the distribution patterns reported here. An expected longer life cycle in the deep sea^73^ may have driven these species into alternative modes of reproduction and dispersal, but the absence of detailed ecological information for Antarctic Philinoidea precludes any conclusions. Alternatively, geological events may explain the current distribution of disjunct species of sea snails across ocean basins^74^.

During the past two decades, an increasing number of molecular studies on different taxa have challenged the three central paradigms of Antarctic benthic lineages^55^, i.e., isolation^64,75^, circumpolarity^15,76^, and eurybathic distributions^16,77^. Our evidence, based on genomic data and novel machine learning approaches, also challenges these long-standing concepts of Antarctic benthic species. Habitat segregation either through shelf refugia or current ecosystem heterogeneity at Antarctic shelf depths may have favored species flocks^59^. Contrarily to the widespread longitudinal distribution of some species^78^, bathymetrically separated distributions are a common phenomenon found in Antarctophilinidae^29^, with higher species diversity and endemism found at shelf depths. Nonetheless, we have to bear in mind the potential biases of sampling efforts across depths, with the lower part of the slope and abyss, seldom explored compared to shallower depths^49^. Shallow-water and slope species are thought to have colonized abyssal depths during the late Mesozoic and Cenozoic epochs^19^. The resulting sinking of cold, saline water adjacent to the Antarctic continent and its subsequent movement northwards at abyssal depths has resulted in colonization from the Antarctic for many invertebrate families and genera. Deep-sea communities seem to harbor less species-level diversity, probably because of more homogeneity in their habitats, compared to the shallow water environments^5^. Strikingly, Antarctica has acted as a center of origin and radiation of certain benthic taxa^19,20^, including Antarctophilinidae mollusks.

## Methods

### Taxon sampling

Antarctic cruises by researchers from multiple institutions were conducted, including the Italian National Antarctic Museum (MNA, Section of Genoa, Italy), the Western Australian Museum (WAM, Perth, Australia), the Benthic Invertebrate Collection at Scripps Institution of Oceanography (SIO-BIC, La Jolla, CA, USA), and the Bavarian State Collection of Zoology (ZSM, Munich, Germany). Sampling stations covered a wide geographical and bathymetric range (10–4550 m) during several cruises (German, Spanish, and US Antarctic Programs) from 1994 to 2017 carried out in the Atlantic sector of the Southern Ocean (Table 1). Distribution maps for the 142 specimens collected, color-coded by species for both continental shelf (Fig. 5a) and slope (Fig. 5b) plains were designed in Arc-GIS 10.3 (Esri, Redlands, CA). Samples were mostly collected by dredging and trawling, but some were collected manually during SCUBA. When possible, specimens were photographed alive on board and preserved in either 70% or 95% EtOH for molecular purposes. Once back in the laboratory, all specimens were photographed dorsally and ventrally using a Keyence VHX-6000 Digital Microscope system at the Museum of Comparative Zoology (MCZ) before dissection.

Additionally, material deposited at the Institute of Paleobiology, Polish Academy of Sciences, of the single philinoid fossil, precisely from Antarctica and originally attributed to *Scaphander* (Scaphandridae)^33^, was morphologically reassessed.

### DNA extraction, library preparation, and sequencing

DNA was extracted from a fragment of the left parapodial lobe using the AutoGenprep 965 Tissue Protocol (AutoGen Inc., Holliston, MA). Initial ‘DNA barcoding’ of all samples was carried out by sequencing a fragment of the mitochondrial cytochrome *c* oxidase subunit I (COI) using primer pair LCO1490 and HCO2198^79^. PCR amplifications were completed in 25-µL reactions with Illustra PuReTaq Ready-To-Go PCR Beads (GE Healthcare, Chicago, IL) with initial denaturation for 5 min at 94 °C, 35 cycles (15 s at 94 °C, 5 s at 48 °C, 15 s at 68 °C), and a final extension step for 7 min at 72 °C. Amplifications were cleaned with incubation of 1 µL ExoSAP-IT (Affymetrix, Santa Clara, CA) and sequencing reactions were performed in 10-µL reactions using BigDye ver. 1 chain-termination chemistry on an ABI3730xl (Applied Biosystems Inc., Foster City, CA). Sequences were edited and aligned with MUSCLE^80^, as implemented in Geneious v. 11.0.3^81^. All sequences were submitted to GenBank (see Table 1 for accession numbers).

Successful DNA extractions were then quantified using a Qubit 2.0 Fluorometer (Invitrogen, Carlsbad, CA), and 100–700 ng of genomic DNA for each sample was used for double-digest restriction site-associated DNA (ddRAD). Libraries were prepared following Peterson et al.^82^ protocol with some modifications, using the enzymes *EcoRI-HF* and *BfaI* (New England Biolabs, Ipswich, MA) for digestion. *Ca.* 50–200 ng of fragmented DNA from each individual was later ligated using the customized P1 and P2 adapters with internal barcodes. Between 15–25 individual samples were then pooled together and size-selected to a range of 350–550 bp using a Blue Pippin (Sage Science Inc., Beverly, MA). Each size-selected pool was amplified through PCR and an Illumina P5 barcode was added using a Phusion High-Fidelity PCR Kit (New England Biolabs). PCRs were conducted with an initial denaturation for 30 s at 98 °C, 10–15 cycles (10 s at 98 °C, 30 s at 65 °C, 30 s at 72 °C), and a final extension for 10 min at 72 °C. Amplified libraries were checked and quantified with a TapeStation 2200 (Agilent Technologies, Carpinteria, CA). Amplified libraries were then cleaned using a ratio of 1:1.5 Agilent beads and quantified using the qPCR Kapa Quantification Kit (Kapa Biosystems, Wilmington, MA). Libraries were multiplexed and paired-end sequenced (150 bp) on an Illumina HiSeq 2500 (Illumina Inc., San Diego, CA) at the Bauer Core Facility, Harvard University (Cambridge, MA).

### Matrix construction

Barcode demultiplexing, quality control, within-sample clustering, and between-pair variant calling were carried out using ipyrad v. 0.7.28^83,84^. Settings and data processing steps are default commonly used with ddRAD data. Only reads with unambiguous barcodes and *Phred* Q scores ≥ 33 were retained, and loci with more than five undetermined bases were additionally discarded. *Maxdepth* was set to 10,000 to avoid uneven sequencing coverage of amplified fragments and highly similar genomic repetitive regions. Thereafter, all individuals from the different libraries were analyzed together, first clustering per locus with a clustering threshold for de novo assembly of 75% (i.e., level of sequence similarity at which two sequences are identified as being homologous, and thus cluster together); additionally, clustering thresholds at 80, 85, and 90% were attempted. Adapters were trimmed and reads < 35 bp discarded. To avoid over-inflation of estimated heterozygosity, we required a minimum of six reads for each cluster during consensus base-calling and up to four shared polymorphic sites per called locus. For finding the consensus loci and clustering across samples we used a minimum number of samples per locus of 50% of total species and a maximum number of SNPs per locus of 20%, only allowing for a 0.05% of ambiguous positions per consensus locus, setting the maximum number of indels to eight (thus filtering out poor final alignments), and a 50% of polymorphic sites per locus. Full datasets were used for the phylogenetic analyses while unlinked SNPs (one randomly selected SNP per locus) were used for the STRUCTURE analysis and variational autoencoder (VAE) plots. Matrix condenser^85^ (available at https://bmedeiros.shinyapps.io/matrix_condenser) was used to visualize the matrix, discard samples with very low coverage (i.e., less than 20% of loci), and construct the final dataset for the downstream analyses. Three different matrices were then constructed for further analyses: Matrix 1, including both the genera *Waegelea* Moles, Avila & Malaquias, 2019 and *Antarctophiline* Chaban, 2016; Matrix 2, including all the species of the genus *Antarctophiline* and with an increased number of shared loci (Fig. S1); and Matrix 3, as a subset of Matrix 2 only including the *A. gibba* (Strebel, 1908) / *A. alata* (Thiele, 1912) species complex. Although depicted in the COI phylogenetic tree, all extractions of *A. easmithi* Moles, Avila & Malaquias, 2019 failed ddRAD library prep, thus they were not included in the matrix construction.

### Phylogenetic and distinct genetic lineages analyses

A phylogenetic tree was inferred on ddRADseq-derived single nucleotide polymorphisms (SNPs) using the maximum likelihood (ML) criterion implemented in RAxML v. 8.2.11^86^ under the GTRGAMMA model. Nodal support was estimated via a rapid Bootstrap analysis (1500 replicates). Bayesian inference (BI) was conducted in MrBayes v. 3.2.6^87^ with the GTR + I + G model^88^. We ran four independent Markov chain Monte Carlo (MCMC) chains for 2 million generations, sampling every 1000 generations and discarding 10% of the trees as burn-in for each MCMC run before convergence. Convergence was achieved when the potential scale reduction factor (PSRF) was close to 1.0 for all parameters. Trees were visualized in FigTree v. 1.4.4^89^ and edited in Adobe Illustrator CC 2018.

Genetic structure and optimal clustering were analyzed in STRUCTURE v. 2.3.4^90^ using matrices with unlinked SNPs for the *Antarctophiline* total dataset (Matrix 2) and the *A. gibba* and *A. alata* species complex (Matrix 3). SNP matrices were run for 1 million generations using an admixture model and 100,000 burn-in on *K* values ranging from 2 to 10 for Matrix 2 and 2–5 for Matrix 3, with eight replicates each. An optimal *K* value was calculated through the Evanno method^32^ in the Structure Harvester Web v. 0.6.94^91^ (http://taylor0.biology.ucla.edu/structureHarvester/). CLUMPAK^92^ (http://clumpak.tau.ac.il/) was used for graphical visualization that was later edited in Adobe Illustrator CC 2018.

To further visualize data and perform clustering on samples we use a VAE^93^ for dimensionality reduction of SNP data. VAEs are a type of machine learning algorithm rooted in Bayesian statistics that relies on neural networks and unsupervised learning to learn a reduced-dimension representation (latent space) of high dimensionality data. This approach allows for easy visualization of the mean and standard deviation of each sample in latent space. The use of VAEs in species delimitation and clustering with genetic data was recently demonstrated by Derkarabetian et al.^36^. The STRUCTURE formatted file was converted to “one-hot encoding” and the VAE was run using the “sp_deli” script (https://github.com/sokrypton/sp_deli) from Derkarabetian et al.^36^. An analysis was run on the full *Antarctophiline* dataset (Matrix 2). However, given potential contamination of sample P70 and issues with sample P49 (see “Results” section), a second analysis was run with these two samples removed. For both datasets, the VAE was run five times and the analysis with the lowest loss (a measure of the difference between input and reconstructed SNPs) was considered optimal. A single estimate of loss was calculated for each analysis by discarding the first 50% of generations as burn-in and calculating the average loss across the second 50% of generations. This average measure of loss for each analysis is akin to the likelihood estimate and burn-in associated with Bayesian phylogenetic analyses. Clustering was performed on the VAE output using only the mean of each sample; the two-dimensional representation (i.e., mean of each sample in latent space) was used as input for multiple clustering methods implemented in R^94^: “partition around medoids” (PAM) clustering using the cluster R package^95^, with the optimal *K* having the highest average silhouette width^96^; PAM clustering with the optimal *K* inferred via the gap statistic with *k*-means clustering using the factoextra R package^97^; and hierarchical clustering with the mclust R package^98^.

## Supplementary Information

Supplementary Informations.

## Data Availability

DNA sequences: GenBank accession numbers are listed in Table 1. Demultiplexed raw reads of ddRADseq-derived data: SRA BioProject PRJNA600882. Matrices and tree files are available in the Harvard Dataverse: https://doi.org/10.7910/DVN/HZJCCS.
