# Supplementary material for: An approach using ddRADseq and machine learning for understanding speciation in Antarctic Antarctophilinidae gastropods
Source: Sci Rep. 2021 Apr 19;11:8473. doi: 10.1038/s41598-021-87244-5 (PMC8055997; doi:10.1038/s41598-021-87244-5)

An approach using ddRADseq and machine learning for understanding speciation in Antarctic Antarctophilinidae gastropods

Juan Moles,[1,*] Shahan Derkarabetian,[1] Stefano Schiaparelli,[2,3] Michael Schrödl,[4,5] Jesús S. Troncoso,[6] Nerida G. Wilson,[7,8] Gonzalo Giribet[1]

[1]Museum of Comparative Zoology, Department of Organismic and Evolutionary Biology, Harvard University, 26 Oxford Street, Cambridge, MA 02138, USA

[2] DiSTAV, University of Genoa, C.so Europa 26, 16132 Genoa, Italy

[3]Italian National Antarctic Museum (MNA, Section of Genoa), Viale Benedetto XV n. 5, 16132 Genoa, Italy

[4]SNSB-Bavarian State Collection of Zoology, Münchhausenstrasse 21, D-81247 München, Germany

[5]Biozentrum Ludwig Maximilians University and GeoBio-Center LMU Munich, Germany

[6] Departamento de Ecoloxía e Bioloxía Animal, Universidade de Vigo, Campus Lagoas-Marcosende s/n, 36200 Vigo, Spain

[7]Collections & Research, Western Australian Museum, Locked Bag 49, Welshpool DC, Perth, WA 6986, Australia

[8]School of Biological Sciences, University of Western Australia, 35 Stirling Hwy, Crawley WA 6009, Australia

*Corresponding author: moles.sanchez@gmail.com / jmoles@g.harvard.edu | orcid.org/0000-0003-4511-4055

ggiribet@g.harvard.edu |orcid.org/0000-0002-5467-8429

Running head: Bathymetric speciation in Antarctic mollusks

Supplementary Material

Table S1. Final Sample stats summary including BioSample Accession name, organism name, number of raw reads, number of reads after filtering steps, the total number of clusters, number of high depth, number of clusters that pass the mindepth thresholds, joint estimation of heterozygosity, error rate, number of consensuses reads, and number of loci in final assembly.

Accession	BioSample.name	BioSample.organism_name	reads_raw	reads_passed_filter	clusters_total	clusters_hidepth	hetero_est	error_est	reads_consens	loci_in_assembly
SAMN13834567	P105	*Antarctophiline alata*	268,792	268,716	47,550	9,874	0.0095	0.0043	8,979	2,859
SAMN13834684	P106	*Antarctophiline alata*	585,281	585,146	80,737	20,203	0.0102	0.0040	18,439	4,510
SAMN13836184	P111	*Antarctophiline* sp. 2	6,709,641	6,708,174	162,577	64,746	0.0063	0.0034	61,108	4,124
SAMN13836185	P112	*Antarctophiline* sp. 2	2,923,780	2,923,085	90,862	39,264	0.0048	0.0032	38,015	3,950
SAMN13835142	P164	*Antarctophiline alata*	2,117,289	2,116,856	100,748	37,354	0.0179	0.0035	32,515	2,308
SAMN13835243	P165	*Antarctophiline alata*	1,450,917	1,450,641	46,722	12,405	0.0151	0.0034	11,013	1,048
SAMN13834750	P175	*Antarctophiline alata*	915,504	915,314	70,953	20,386	0.0089	0.0047	18,743	4,740
SAMN13834902	P177	*Antarctophiline alata*	127,337	127,308	34,678	4,272	0.0102	0.0052	3,803	1,547
SAMN13833096	P286	*Antarctophiline amundseni*	279,789	279,735	57,553	10,114	0.0087	0.0052	9,083	1,669
SAMN13833226	P287	*Antarctophiline amundseni*	4,723,374	4,722,315	161,100	88,485	0.0154	0.0046	78,117	3,609
SAMN13833404	P304	*Antarctophiline* sp. 1	647,028	646,896	61,966	25,336	0.0099	0.0040	23,678	2,890
SAMN13833597	P322	*Antarctophiline* sp. 1	705,578	705,428	77,040	20,826	0.0107	0.0044	19,268	2,751
SAMN13836178	P355	*Antarctophiline amundseni*	2,634,923	2,634,393	119,243	40,457	0.0063	0.0038	38,596	3,572
SAMN13835028	P42	*Antarctophiline alata*	2,426,489	2,425,969	111,561	40,795	0.0064	0.0033	39,069	5,281
SAMN13834180	P45	*Antarctophiline alata*	1,071,817	1,071,581	62,505	29,257	0.0072	0.0036	27,524	4,257
SAMN13832947	P48	*Antarctophiline amundseni*	2,618,759	2,618,176	108,656	38,180	0.0064	0.0036	36,490	3,479
SAMN13833791	P49	*Antarctophiline gibba*	113,874	113,847	31,199	3,601	0.0110	0.0051	3,188	1,055
SAMN13833939	P50	*Antarctophiline gibba*	1,491,349	1,491,008	80,176	29,853	0.0060	0.0033	28,650	4,911
SAMN13835366	P52	*Antarctophiline* sp. 3	1,518,912	1,518,613	80,942	30,510	0.0064	0.0035	29,176	5,223
SAMN13835536	P53	*Antarctophiline* sp. 3	770,963	770,821	65,979	22,155	0.0062	0.0038	21,095	4,958
SAMN13836079	P58	*Antarctophiline* sp. 3	8,578,103	8,576,203	69,627	24,626	0.0095	0.0040	22,903	4,225
SAMN13835769	P65	*Antarctophiline* sp. 3	578,660	578,539	61,627	20,605	0.0076	0.0039	19,250	4,826

SAMN138 36091	P66	*Antarctophiline* sp. 3	401,171	401,083	22,556	9,860	0.0071	0.0038	9,298	2,481
SAMN138 36069	P67	*Antarctophiline* sp. 3	1,875,0 38	1,874,595	61,670	34,872	0.0067	0.0034	33,258	5,223
SAMN138 36077	P70	*Antarctophiline* sp. 3	703,246	703,087	19,484	5,979	0.0092	0.0065	5,523	1,132
SAMN138 35766	P73	*Antarctophiline* sp. 3	488,301	488,217	41,429	9,216	0.0094	0.0043	8,439	2,980
SAMN138 36080	P74	*Antarctophiline* sp. 3	177,742	177,712	15,959	6,647	0.0076	0.0037	6,224	1,602
SAMN138 36078	P75	*Antarctophiline* sp. 3	5,444,9 21	5,443,755	28,255	5,785	0.0104	0.0061	5,376	1,350
SAMN138 35767	P81	*Antarctophiline* sp. 3	874,963	874,769	57,854	15,482	0.0087	0.0042	14,292	3,994
SAMN138 35768	P82	*Antarctophiline* sp. 3	515,862	515,748	31,136	3,941	0.0115	0.0070	3,482	1,291
SAMN138 36110	P86	*Antarctophiline* sp. 3	136,161	136,127	35,238	3,545	0.0150	0.0050	3,041	973
SAMN138 35647	P87	*Antarctophiline* sp. 3	418,132	418,050	50,018	14,566	0.0082	0.0040	13,499	4,122
SAMN138 34249	P91	*Antarctophiline alata*	385,772	385,688	44,088	10,392	0.0089	0.0043	9,487	3,173
SAMN138 34431	P92	*Antarctophiline alata*	165,315	165,270	36,060	6,598	0.0081	0.0043	6,064	2,356
SAMN138 34362	P94	*Antarctophiline alata*	1,065,8 64	1,065,633	88,241	27,784	0.0074	0.0039	26,132	5,225

Figure S1. Occupancy matrix for the *Antarctophiline* dataset (Matrix 2), with 5411 loci and 41.6% missing data. Loci and species are sorted with the best sampling on the upper left. Green cells indicate loci present for each species.

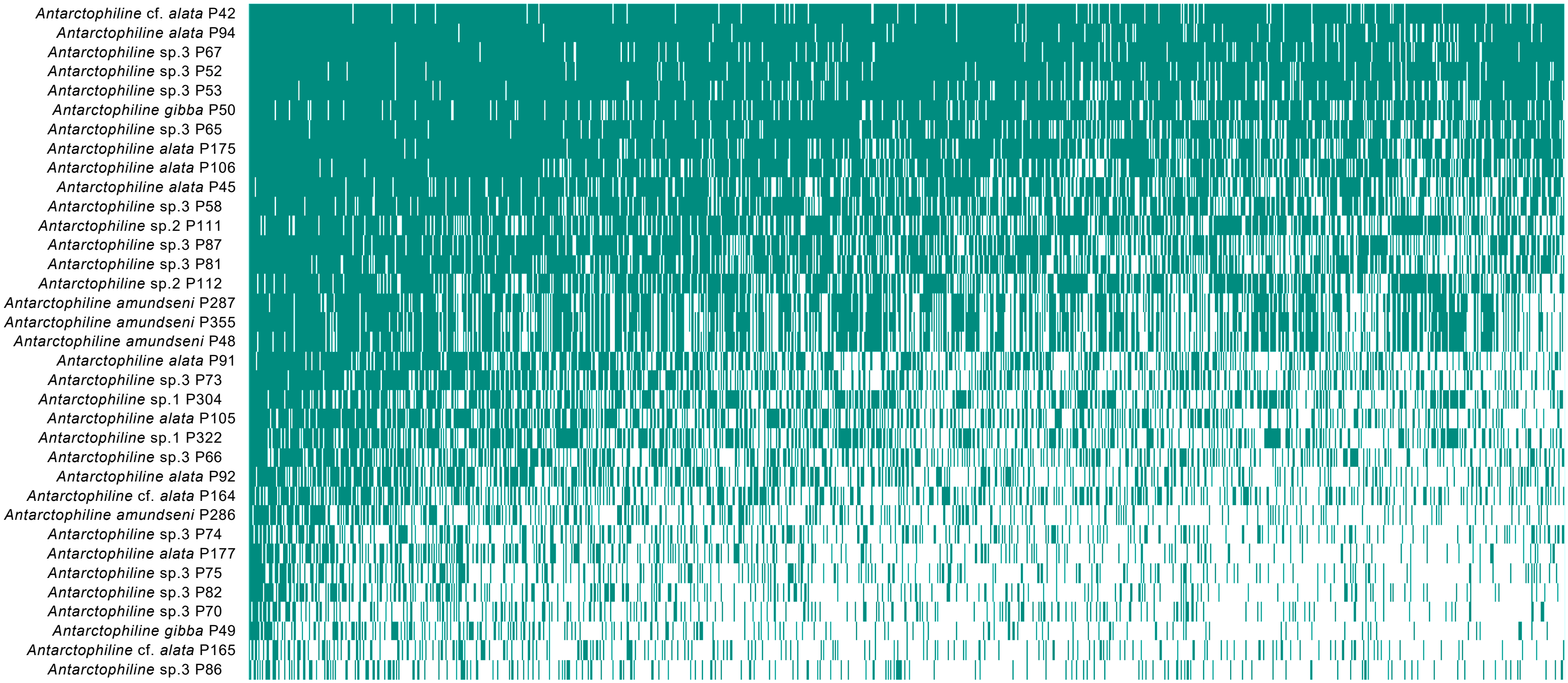

Figure S2. Phylogenetic relationships of antarctophilinids based on ddRADseq data of Matrix 1 and a maximum likelihood analysis (see Material and Methods). The tree is rooted with the sister group to *Antarctophiline*, *Waegelea antarctica*.

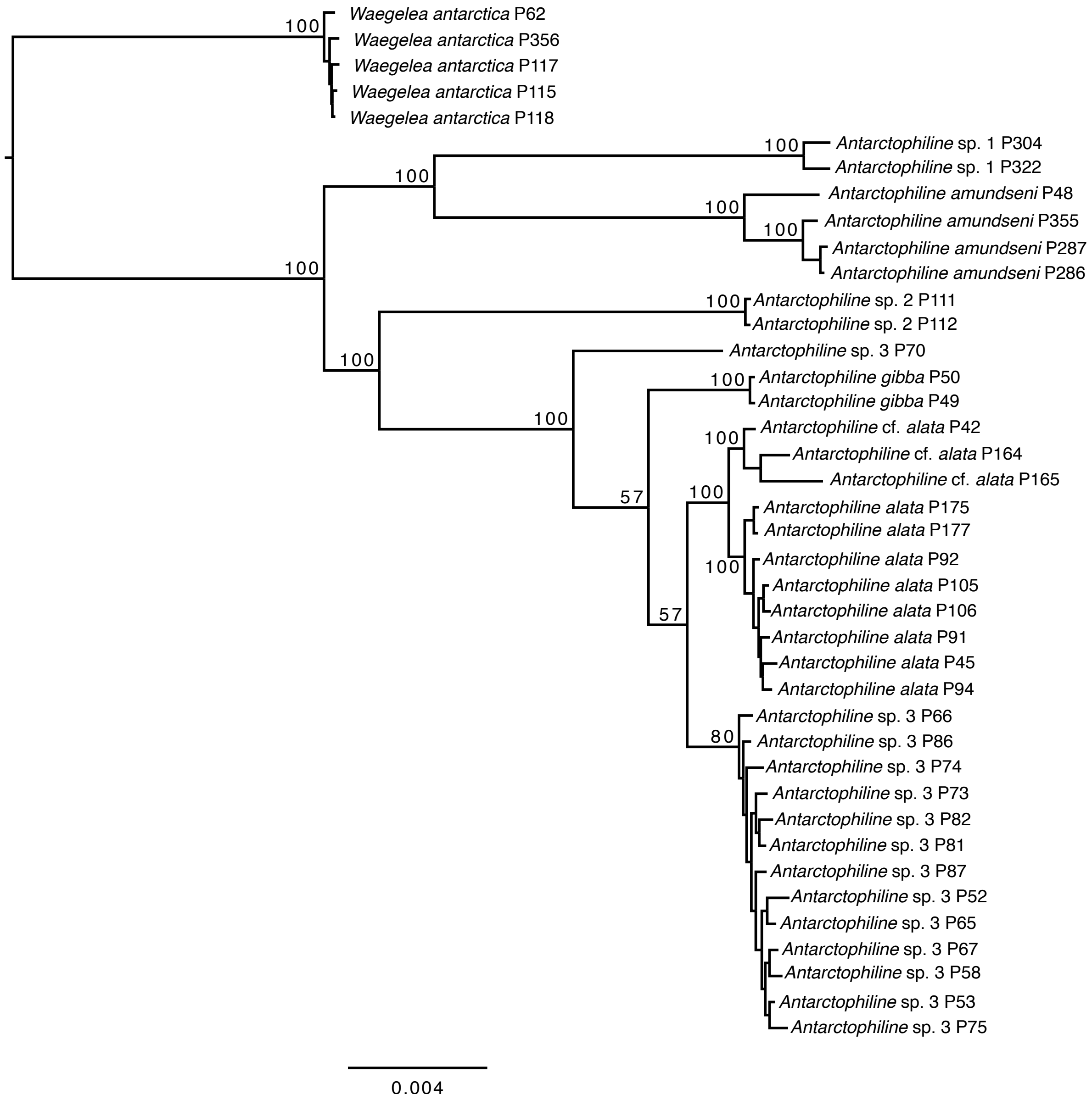

Supplement: Supplementary file 1 — Supplementary Informations. [file 41598_2021_87244_MOESM1_ESM.pdf]
